# Magnetic and structural properties of glass-coated Heusler-type microwires exhibiting martensitic transformation

**DOI:** 10.1038/s41598-017-19032-z

**Published:** 2018-01-12

**Authors:** A. Zhukov, M. Ipatov, J. J. del Val, V. Zhukova, V. A. Chernenko

**Affiliations:** 1 Dpto. de Fís. Mater., UPV/EHU, San Sebastián, 20018 Spain; 2 Dpto. de Física Aplicada, EUPDS, UPV/EHU, 20018 San Sebastian, Spain; 3 IKERBASQUE, Basque Foundation for Science, 48013 Bilbao, Spain; 4 BCMaterials & University of Basque Country (UPV/EHU), Bilbao, 48080 Spain; 5 Institute of Innovative Research (IIR), Tokyo Institute of Technology, 226-8503 Yokohama, Japan

## Abstract

We have studied magnetic and structural properties of the Heusler-type Ni-Mn-Ga glass-coated microwires prepared by Tailor-Ulitovsky technique. As-prepared sample presents magnetoresistance effect and considerable dependence of magnetization curves (particularly magnetization values) on magnetic field attributed to the magnetic and atomic disorder. Annealing strongly affects the temperature dependence of magnetization and Curie temperature of microwires. After annealing of the microwires at 973 K, the Curie temperature was enhanced to about 280 K which is beneficial for the magnetic solid state refrigeration. The observed hysteretic anomalies on the temperature dependences of resistance and magnetization in the as-prepared and annealed samples are produced by the martensitic transformation. The magnetoresistance and magnetocaloric effects have been investigated to illustrate a potential technological capability of studied microwires.

## Introduction

Stoichiometric and non-stoichiometric X_2_YZ (X and Y are transition metals and *Z* is the main group element) Heusler alloys exhibiting first order martensitic transformation (MT) have gained growing attention due to a number of versatile properties promising for applications. They show the related-to-MT prototypical magnetic shape-memory (MSM) effect, a remarkable magnetocaloric effect (MCE), large manetoresistance (MR) influenced by the strong magnetoelastic coupling and concurrent ferro - antiferromagnetic interactions^1–3^.

The most important applied aspect of MSM materials is the magnetic solid state cooling presenting potentially better energy efficiency than the conventional refrigeration techniques. One of the ways to improve an efficiency of a refrigeration machine is an increase of the surface-to-volume ratio of working material needed to enhance the heat exchange rate. Therefore, the development of low-dimensional Heusler alloys (e.g., thin films or thin wires) presenting MCE effect is highly desirable^1,4–9^.

Heusler compounds, including Ni-Mn-Ga alloys, are brittle. This prevents their preparation in a low dimensional form by the conventional metallurgical techniques^1^. Consequently, a considerable attention is continuously paid to the development of novel fabrication methods of Heusler alloys in the form of thin films or wires^5–9^.

Particularly, a Taylor-Ulitovsky technique was adopted for a fabrication of the quasi-unidimensional Ni-Mn-In, Ni-Mn-In-Co and Ni-Mn-Ga MSM Heuslers in the form of a metallic microwire covered by a flexible glass coating^5–7^. This technique involves a rapid quenching from the melt. The thin glass-coated microwire has a (nano-)microcrystalline structure^4–7,10–12^. Typical diameters of the metallic nucleus in such microwires are ranging between 1 and 40 μm, the thickness of flexible and insulating glass-coating is between 0.5 μm and 20 μm. The length of wire can be up to 10 km which means good mechanical properties of the product.

Alongside disordered structure, one of the main problems of the glass-coated microwires is the appearance of large internal stresses ranging from 100 to 1000 MPa, resulting from the rapid simultaneous solidification of metallic nucleus with glass-coating having essentially different thermal expansion coefficients. These internal stresses are distributed in a complicated manner within the microwires^5–7,10–12^. These stresses and disordered crystalline structure can impede the martensitic transition. Whereas in much thicker Heusler wires without coating it was not big issue to activate MT (see^13,14^ and references therein), we had encountered difficulties to get MT in our recent reports on the ultrathin glass-coated Heusler wires^5–7,10^. It is worth noting that the thick wires do not provide good enough mechanical properties and enhanced flexibility needed for a broad range of applications^15–17^, as thin and long glass-coated wires do.

Thus, the proper recrystallization process and stress relaxation, alongside the alloy composition, are needed to enable observation of MT in thin microwires. In the present paper we report our latest results on the preparation, processing and full characterization of MSM Ni-Mn-Ga microwires. The results show that microwires exhibit MT and relevant magnetic properties ensuring the advanced actuation and caloric properties of these materials.

## Results and Discussion

### Magnetization and transformation behaviours

**Figure 1:**
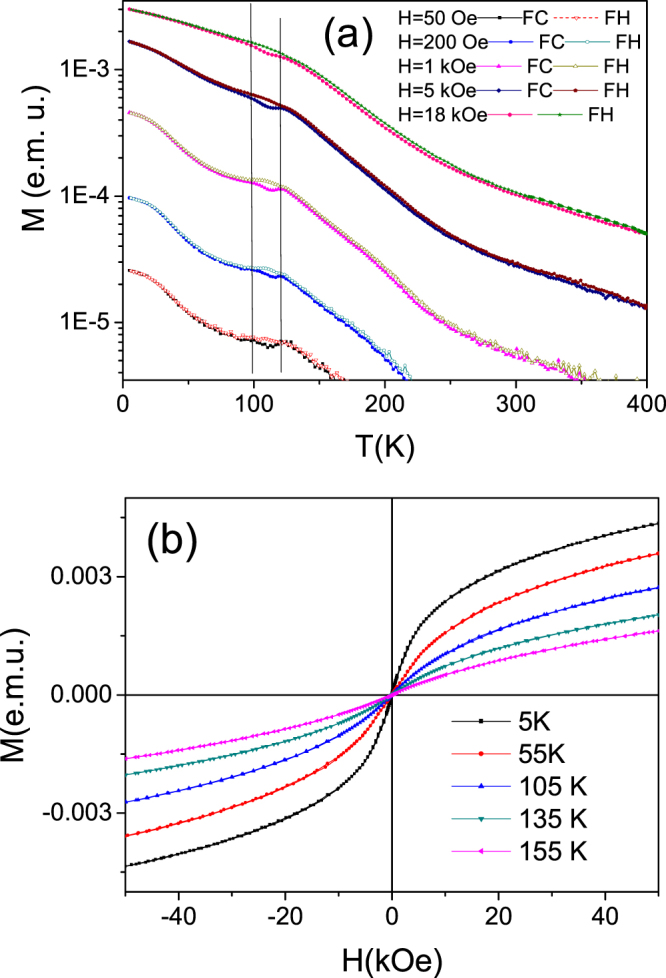
Temperature dependence of magnetic moment measured for as-prepared Ni-Mn-Ga microwire (**a**) and hysteresis loops measured at different temperatures (**b**).

As-prepared microwires exhibit quite a weak magnetism in whole temperature range. Temperature dependence of magnetic moment, *M(T)*, in Fig. 1a shows a non-monotonic decrease during heating due to the approach to fully paramagnetic state and a small and narrow hysteretic anomaly between field cooling (FC) and field heating (FH) curves at different magnetic fields, H. This small hysteretic anomaly on *M(T)* dependence in the temperature interval of 100–120 K is observed at all H-values and can be attributed to the presence of martensitic transformation in the wires. Since the difference in the magnetization between martensite and austenite is small, no visible shift of MT temperature under H is found.

The *M(T)* dependences in Fig. 1a reflect a nonuniform magnetic character of the as-received wire produced by the atomic disorder and magnetic clustering. Despite the smeared shape of *M(T)* curves, the Curie temperature of as-received sample, *T*_*C(as-rec)*_, can be located at about 165 K at 50 Oe or so. A strong influence of magnetic field on *T*_C(as-rec)_ and on the absolute M-values is typical for the non-uniform magnetic systems containing ferromagnetic clusters in paramagnetic matrix (see, e.g.^18^,). Such systems show also signs of superparamagnetism as it follows from a comparison of *M(H)* curves obtained for as-received Ni-Mn-Ga/MgO thin films^19^ and curves in Fig. 1b. In both cases *M(H)* curves do not show both the saturation and coercive force.

**Figure 2:**
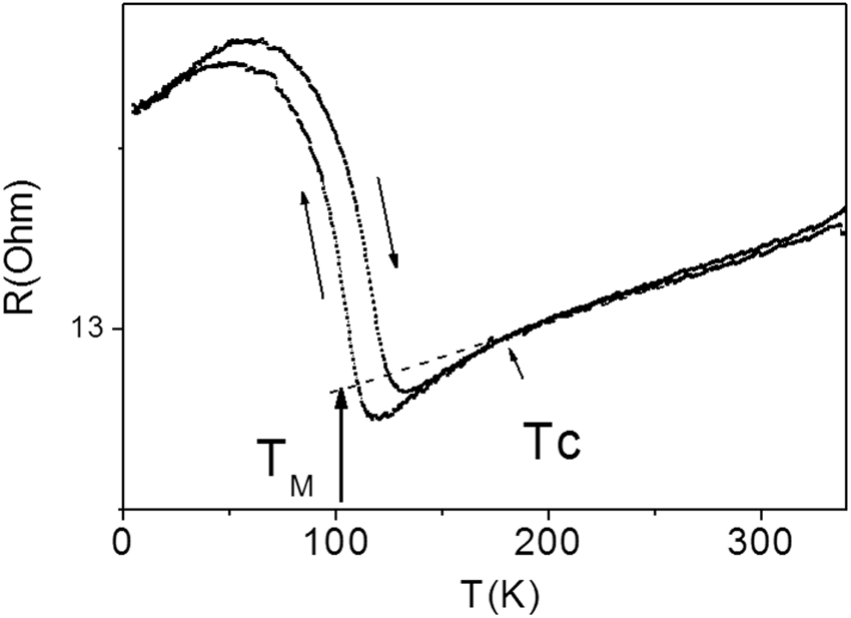
Temperature dependence of resistance, *R*, for as-prepared Ni-Mn-Ga microwire measured at zero field.

Temperature dependence of resistance, *R(T)*, in Fig. 2 shows the typical for Ni-Mn-Ga alloys hysteretic anomaly and kink as the unequivocal signatures of MT and Curie point of the cubic austenite, respectively^8^. Whereas *M(T)* and *M(H)* curves in Fig. 1 reflect the magnetic states in the wire, *R(T)* behavior in Fig. 2 is mainly affected by a structural condition of the austenitic matrix. As already mentioned, the magnetic characteristics of as-received wire are presumably governed by an ensemble of ferromagnetic clusters which is very sensitive to the external magnetic field and much less susceptible in our case to MT of the matrix. In fact, the latter point is a reason of the very small, about 25 K, transformation range manifested through *M*(*T*) curves, in contrast to the total width of MT interval in *R*(*T*) curve, equal to about 80 K. During cooling both *R*(*T*) and *M*(*T*) shows start of MT at about 120 K and within 10 K a significant amount of martensitc phase is formed. The reverse MT is usually more smeared than the forward one. On heating *R*(*T*) dependence shows the start of reverse MT at about 60 K, while *M*(*T*) exhibits change only in the range between 95 K and 105 K, where the steep part of heating part of *R*(*T*) anomaly occurs. Thus, the temperature corresponding the middle point of cooling part of the anomaly on *R(T)* curve, equal to 100 K, can be taken as the characteristic MT temperature, *T*_*M(as-rec)*_, for as-received wire. At the same time, both *R*(*T*) and *M*(*T*) curve shows similar value of Curie temperature of the cubic austenite,*T*_*C(as-rec)*_, close to about 165 K.

**Figure 3:**
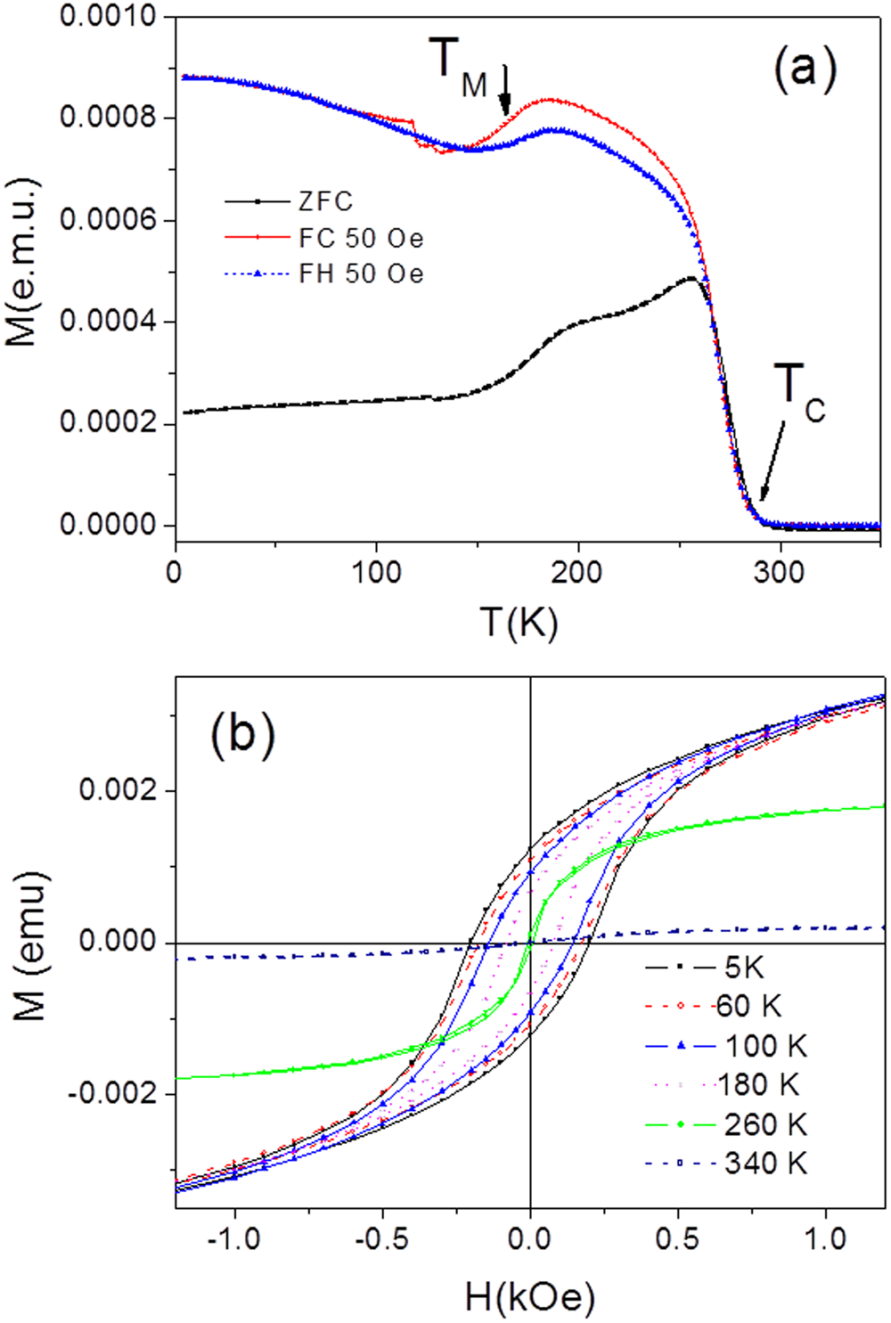
Temperature dependence of magnetic moment (**a**) and hysteresis loops (**b**) measured for the NiMnGa glass-coated microwires annealed at 973 K for 1 h.

Annealing of the samples resulted in a drastic change of magnetic properties and MT behavior. Particularly, a ferromagnetic ordering with Curie temperature, *T*_*C*_, near room temperature is observed (see Fig. 3). While annealing shifted *T*_C_ to high temperatures by about 100 K, the MT temperature was increased by about 60 K (cf. Figs 1a and 3a). The temperature dependencies in Fig. 3a show clearly that Ni-Mn-Ga microwire during cooling exhibits, first, a ferromagnetic ordering of austenite at *T*_*C*_ and then martensitic transformation at *T*_*M*_ (see also refs^20–23^). The MT in Ni-Mn-Ga Heusler alloys is a displacive phase transformation from a high symmetry cubic austenite to a low symmetry martensite accompanied by temperature hysteresis due to its first-order nature. Thus, the hysteresis observed between heating and cooling *M*(*T*) curves (Fig. 3a) is an unequivocal signature the first-order martensitic phase transition in microwires. Due to the hysteresis, the temperature dependence of the volume fraction for each of the two phases could be different for the heating and cooling ramps.

The transitions temperatures *T*_C_ and *T*_*M*_ are basically predetermined mainly by the chemical composition of Ni-Mn-Ga alloy also characterized by the valence electron concentration per atom, e/a^22,24,25^. For Ni_59.2_Mn_12.2_Ga_28.6_ alloy with e/a = 7.63, *T*_*C*_ > *T*_*M*_ should be held, that is in an agreement with observed in Figs 1a and 3a anomalies sequence on the *M(T)* dependences^20–22^.

Figure 3b depicts magnetic hysteresis loops for annealed sample measured at different temperatures representing different magnetization process in the paramagnetic austenite (*T* = 340 K), ferromagnetic austenite (*T* = 260 K) and ferromagnetic martensite (*T* < 180 K). These magnetic states became well distinguished owing to the mechanism of the thermally induced increment of atomic order giving rise to more homogeneous magnetic state of the austenitic matrix. The *T*_C_ values observed in Figs 1a and 3a are still smaller than the value corresponding to *e*/*a* ≈ 7.63 in the phase diagram in ref.^22^, equal to about 370 K. This fact can be accounted by for the essentially reduced amount of the Mn atoms per unit cell which are main carriers of the localized magnetic moments in the NiMn-based Heusler alloys.

**Figure 4:**
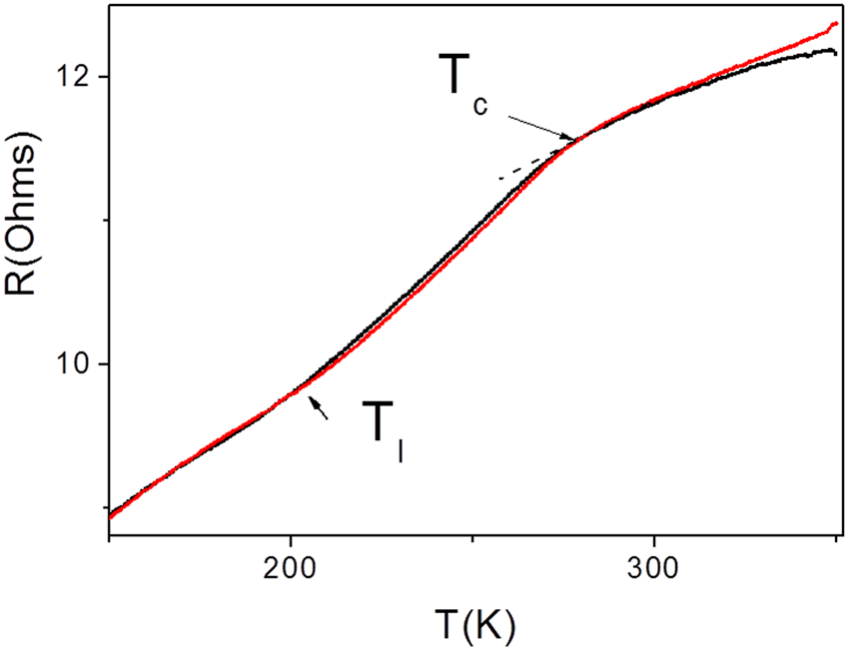
Temperature dependence of resistance for annealed at 973 K for 1 h Ni-Mn-Ga microwires measured at *H* = 0.

The *R(T)* dependence for annealed wire depicted in Fig. 4 confirms the value of Curie temperature, *T*_*C*_ = 280 K, determined from *M(T)* curve (Fig. 3a), but does not show anomaly in the temperature range where MT is expected to occur based on the data of Fig. 3a, i.e., at 160 K. Instead, a curve deflection at the temperature T_I_, where the upward change of the curvature occurs, is observed. This temperature can be attributed to the transformation temperature into intermediate phase which is typically observed in the bulk Ni-Mn-Ga alloys (see, e.g.^26^,). It should be mentioned that disappearance of *R(T)* anomaly at MT is typical for the bulk polycrystalline low-temperature Ni-Mn-Ga alloys with similar e/a values (see ref.^22^).

Thus, the aforementioned transformation behaviors of the Ni-Mn-Ga microwires, observed in this work by *M(T)* and *R(T)* measurements, have been understood by comparing with similar results for the bulk alloys well-known from the literature.

It is worth noting that the temperature dependence of resistivity in Ni-Mn-Ga alloys is considerably affected by the valence electron concentration per atom, e/a^22^. Similar decreasing of *dR/dT* above *T*_*c*_ is reported for bulk Ni-Mn-Ga alloys with *T*_*M*_ < *T*_*C*_ (*e*/*a* < 7.7) and discussed in terms of a difference in the magnetic scattering of electrons above and below *T*_*C*_^22^.

A considerable effect of annealing on the *M(T)* dependencies, leading to a homogenization of the magnetic state in the wire, must be attributed to the improvement of the atomic and crystallographic order and internal stresses relaxation. As mentioned above, one of the main peculiarities of the glass-coated microwires is the fabrication method involving simultaneous rapid solidification from the melt of metallic alloy and glass-coating with quite different thermal expansion coefficients^11,12,27^. Similarly to the studied Ni-Mn-Ga microwires, a restoring of a ferromagnetic state has been reported after annealing of Ni-Mn-Ga thin films presenting the paramagnetic behavior in a wide temperature range in as-prepared state^8^.

### Magnetoresistance

**Figure 5:**
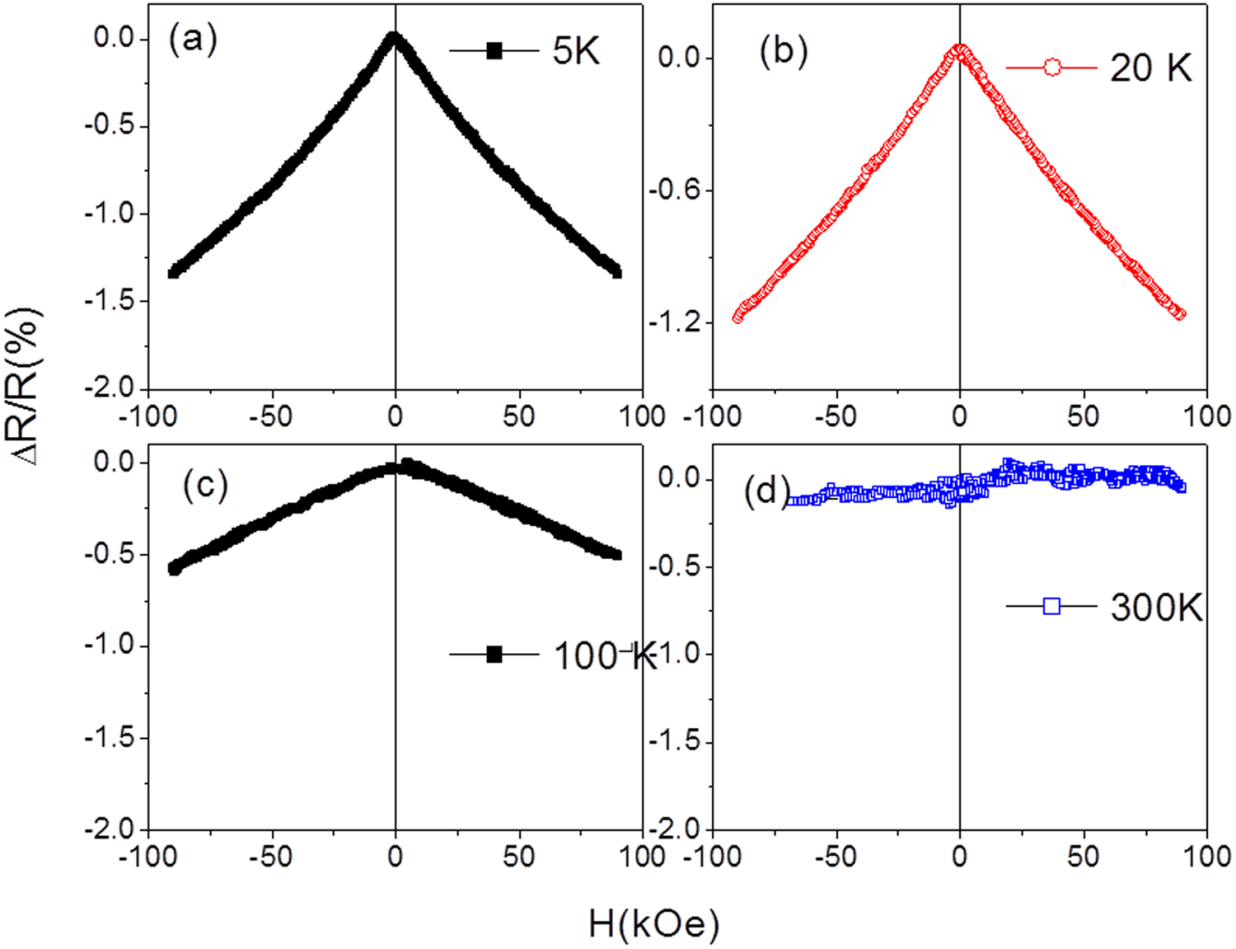
Δ*R*/*R* dependence for as-prepared Ni-Mn-Ga microwires measured at different temperatures.

The magnetic field dependences of electrical resistance, *R(H)*, were recorded and then magnetoresistance, *ΔR/R*, was evaluated. As can be appreciated from Fig. 5, as-prepared Ni-Mn-Ga microwire demonstrates a magnetoresistance effect (MR) at low temperatures (5, 20 and 100 K), whereas no MR was observed in a non-magnetic phase at 300 K.

The decrease of resistivity with magnetic field is typical for a giant magnetoresistance (GMR) effect. The origin of the GMR effect is commonly attributed to the spin-dependent scattering of the conduction electrons at the interfaces between single domain nanograins and nonmagnetic matrix as well as within the magnetic granules^28–31^. On the other hand, the magnetically inhomogeneous materials can also present a considerable MR effect. MR effect was reported in materials consisting of magnetic clusters within a non-magnetic matrix^32^ or precipitates formed by a spinodal decomposition^33^.

In classical materials showing GMR effect (either multilayers or granular alloys), MR can be saturated under a sufficiently large magnetic field when ferromagnetic alignment is achieved. In contrast, in heterogeneous magnetic systems, like alloys containing ferromagnetic clusters embedded in non-magnetic matrix or spin glasses, MR cannot be saturated by the magnetic field due to the lack of fully parallel magnetic moments alignment. The common feature of all of the materials presenting MR effect is the existence of a conducting medium with magnetic inhomogeneities on the scale of the electron mean-free path^28–31^.

In our case the MR can be related to some structurally triggered magnetic inhomogeneities or clusters in the microwire related to the preparation method.

**Figure 6:**
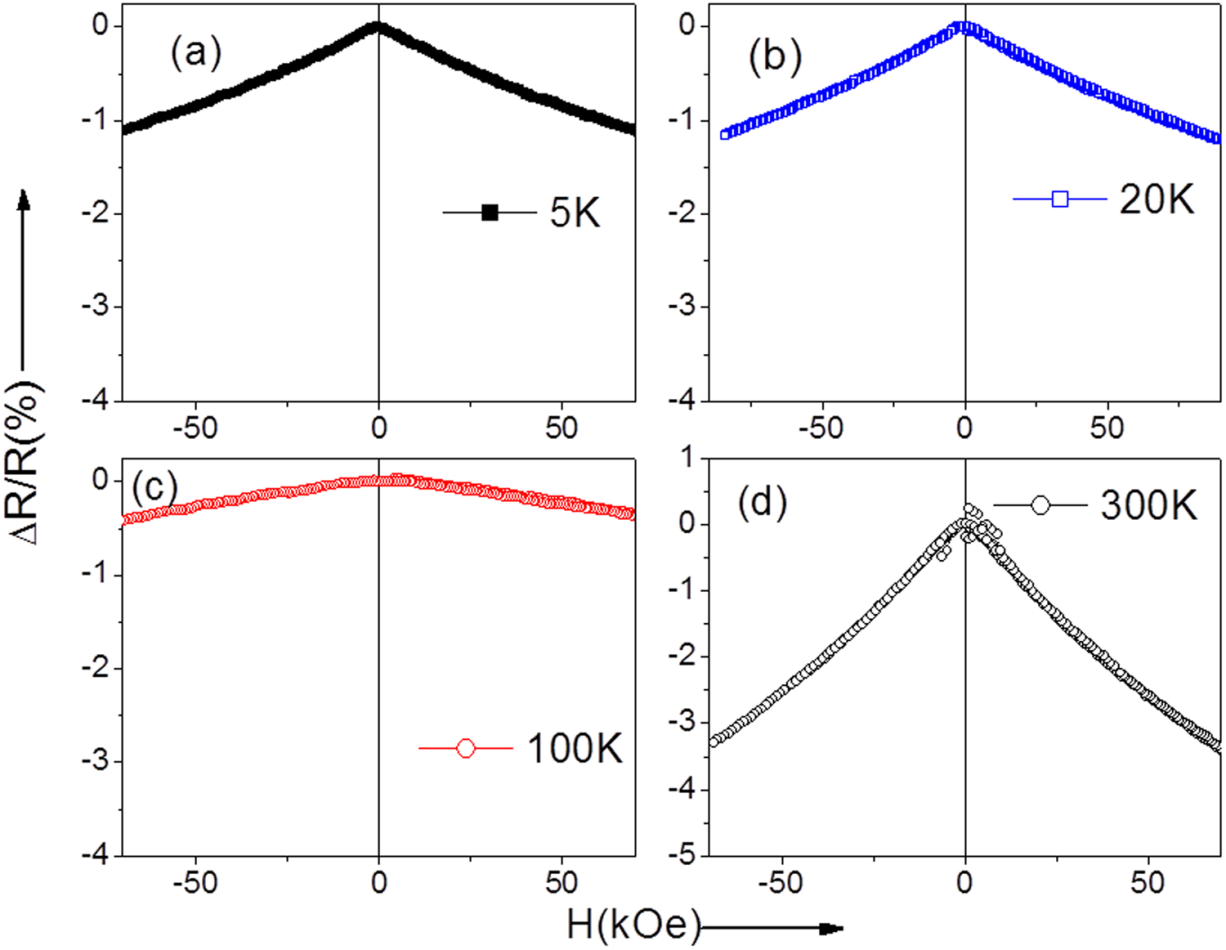
Δ*R/R* dependences for annealed at 973 K for 1 h Ni-Mn-Ga microwires measured at different temperatures.

Similarly to the as-prepared sample, we have measured Δ*R/R* dependences at different temperatures in Ni-Mn-Ga glass-coated microwires annealed at 973 K for 1 h. Figure 6 shows that at low temperatures (5 K and 20 K), a decrease of resistivity with magnetic field typical for giant magnetoresistance (GMR) effect and similar to the as-prepared Ni-Mn-Ga glass-coated microwires is observed. Much higher MR effect is observed in the vicinity of Curie temperature at 300 K, where the enlarged magnetic fluctuations are expected.

### Magnetocaloric effect

**Figure 7:**
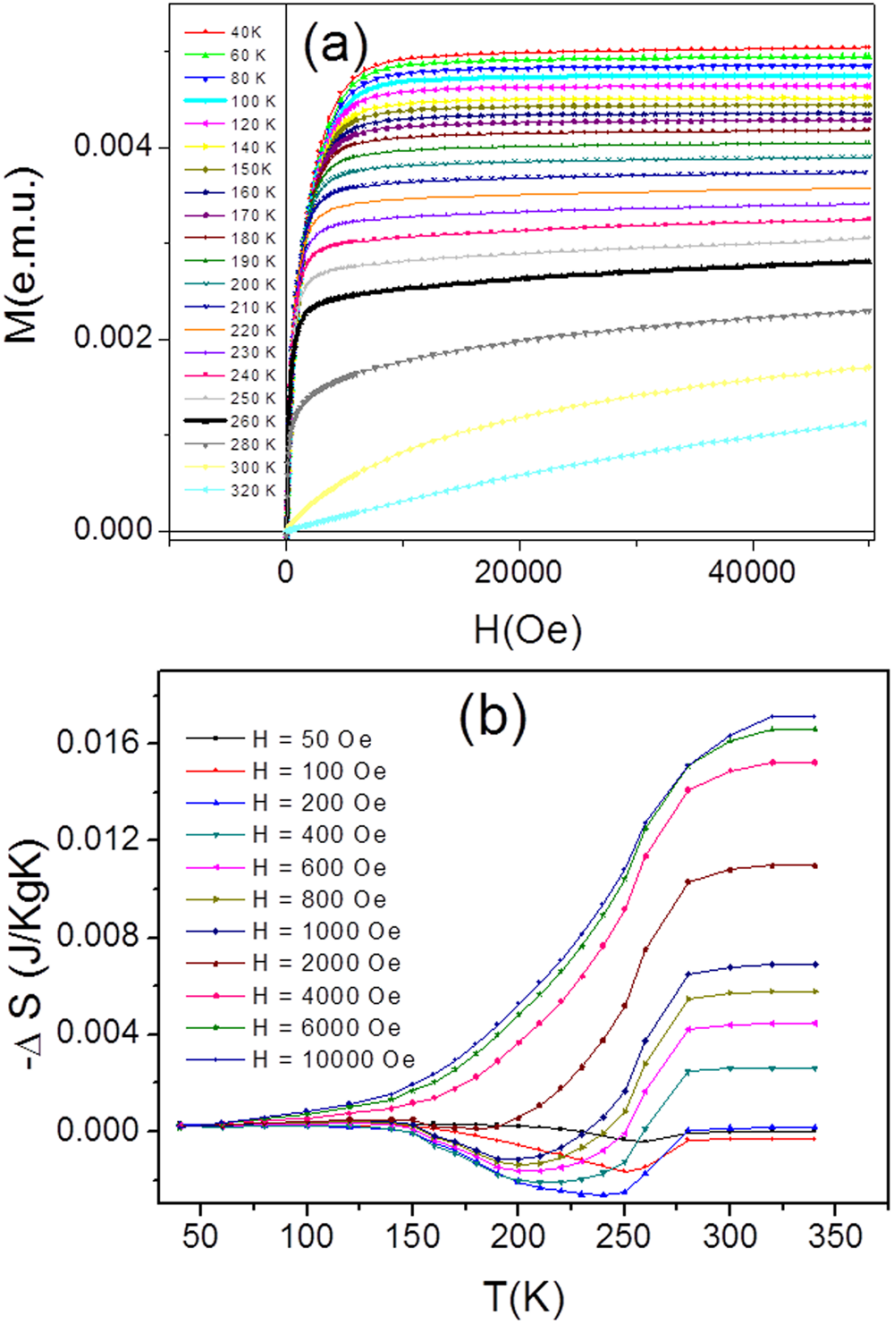
Magnetization curves of Ni-Mn-Ga microwires annealed at 973 K measured at different temperatures (**a**); and calculated dependencies of the field-induced entropy change (**b**).

Magnetocaloric effect was studied by the indirect method. Figure 7a shows the virgin magnetization curves *M(H,T)* measured around the ferromagnetic transition of the sample annealed at 970 K(1 h). From these magnetization curves, measured at different temperatures, we have calculated the magnetic field induced entropy change, *ΔS*, following the procedure described elsewhere^3,34^.

Figure 7b shows that *ΔS(T)* exhibits a tendency to a maximum at *T*_C_ point equal to about 0.2 J/KgK, which is slightly below than that found in other low-dimensional Heusler-type materials showing a ferromagnetic transition^7^. Like in the case of Ni-Mn-Ga film^9^, this value can be multiplied by one or two orders of magnitude if wire is made of Ni-Mn-Ga alloy where *T*_C_ is merged with MT. Tiny *ΔS(T)* minimum at the small fields is explained by the negative difference in the magnetization between martensite and austenite due to the large uniaxial magnetic anisotropy of martensite.

Resuming, we have demonstrated that after appropriate post-processing the thin glass-coated microwires of Ni-Mn-Ga alloy exhibit a much more favourable combination of MT and the magnetic properties, among them nearly ambient Curie temperature, than in as-received state. The large MR effect was measured. The significant MCE was estimated being potentially much enlarged after merging MT with *T*_C_. The observation of the martensitic transformation is confirmed by various phenomena, such as standard hysteretic anomalies on the temperature dependences of the physical properties.

Such thin wires show great potential in a number of applications, particularly, in the magnetic refrigeration^4^.

## Methods

Glass-coated Ni-Mn-Ga microwires with a metallic nucleus diameter of *d* ≈ 22 μm and total diameter of *D* ≈ 62 μm, respectively, have been prepared using master alloy with a nominal composition of Ni_50_Mn_25_Ga_25_ (at.%) by a Taylor-Ulitovsky technique described elsewhere^5–12^

The as-prepared microwires were annealed in a conventional furnace in order to release the internal stresses and achieve a more ordered structural and magnetic state. Previously we usually used annealing temperatures in the range of 773–823 K^5–7,10,12^, but we did not observe any evidence of MT either in as-prepared or annealed Ni-Mn-Ga microwires^5–7^. Therefore in the present work, in order to facilitate more homogeneous state and reduce internal stress the higher values of annealing temperatures, between 923 K and 973 K, were chosen.

**Figure 8:**
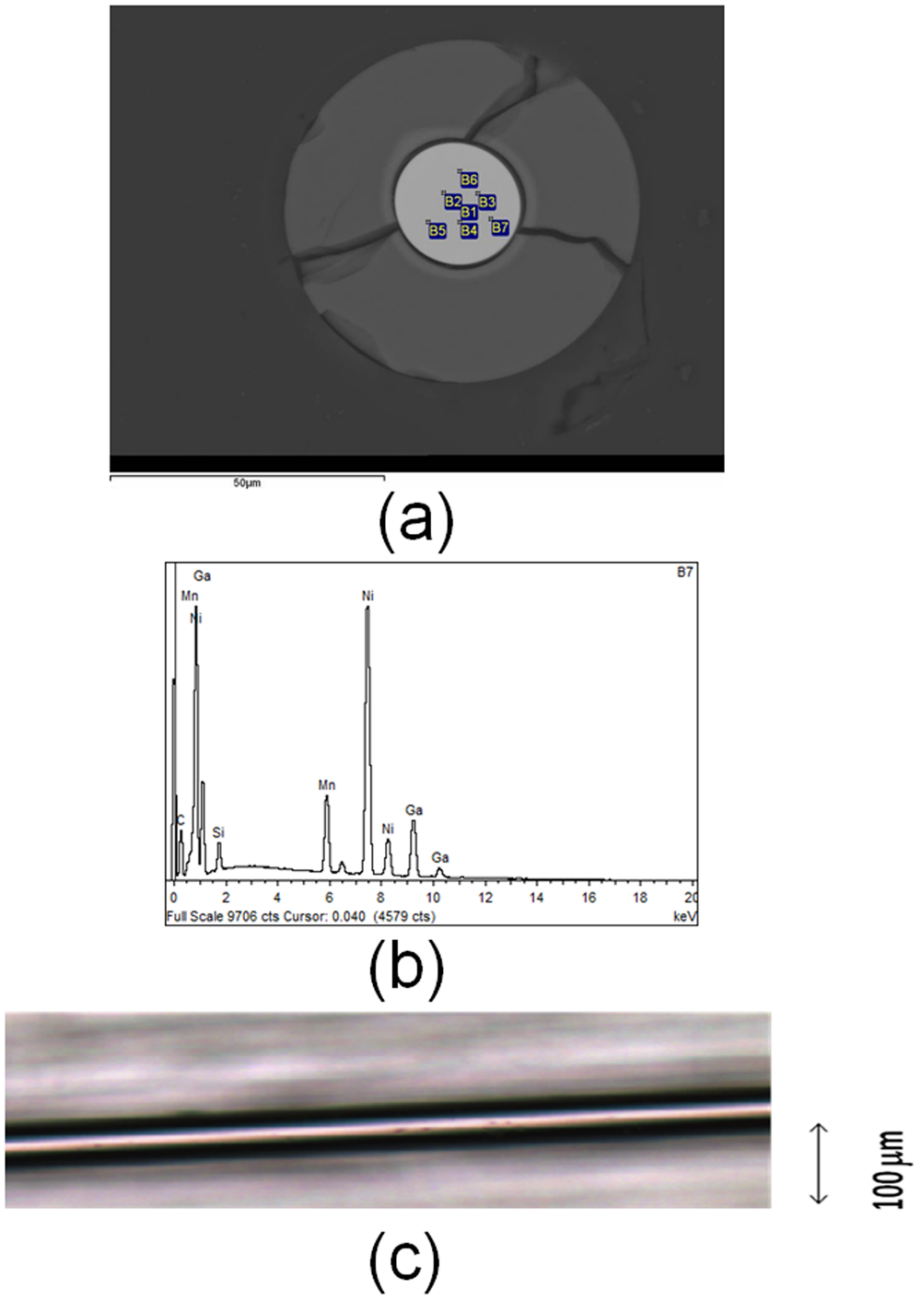
Image of the cross-section (**a**); EDX spectra of one of the points, B7, where the chemical composition was determined (**b**) and optical microscopy image (**c**) of the Ni-Mn-Ga microwire.

The cross-section images of the sample placed inside the epoxy and mechanically polished were obtained using the scanning electronic microscopy (Fig. 8a). The inner part of the image corresponds to the metallic nucleus. The outer shell is a glass-coating with thickness of about 20 μm. The metallic nucleus presents quite regular circular cross-section with a diameter (including the interface layer) of about 22 μm (Fig. 8a). The black lines in the Fig. 8a correspond to the cracks that arose during the mechanical polishing of the sample edge.

The chemical composition of the metallic nucleus evaluated using EDX is quite homogeneous indicating almost the same composition in different sites of the cross section (marked as B1-B7). The metallic nucleus chemical composition evaluated using EDX (see Fig. 8b where the EDX spectra taken in point B7 of the metallic nucleus indicated in Fig. 1a is shown) is Ni_59.2_Mn_12.2_Ga_28.6_ (at.%) with uncertainty of less than 1.0 at.%_._ This difference with the nominal composition indicates that Mn was partially evaporated during the microwire casting.

We used the microscope Axio Scope A1 for the metallographic studies of glass-coated microwires. From optical images we can deduce that prepared microwire presents rather homogeneous metallic nucleus and total diameters (see Fig. 8c).

Structural and phase states have been studied using a BRUKER (D8 Advance) X-ray diffractometer. *CuKα* (λ = 1.54 Å) radiation was used in all measurements.

**Figure 9:**
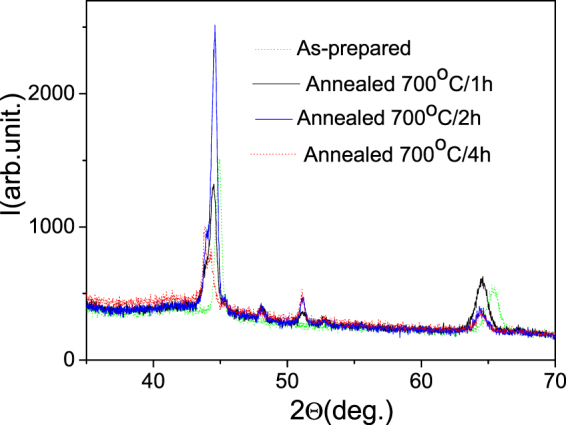
XRD patterns of as-prepared and annealed at 973 K for different times Ni-Mn-Ga microwires.

X-ray diffraction patterns at room temperature for the as-prepared and annealed samples are shown in Fig. 9. Both types of samples are polycrystalline with the small grains (Scherrer formula provides an average size of the crystallinity domains of around 25–30 nm). Likewise in the case of Ni-Mn-Ga thin films^35^, a reasonable interpretation of the crystallographic state of the wires can be suggested taking into account the whole set of experimental data obtained in this work. According to the measured physical properties described in the following sections, all wires are in the austenitic cubic phase at room temperature. In terms of cubic L2_1_-ordered Heusler structure, the peaks near 44 deg. and 64 deg. in Fig. 2 can be attributed to the main characteristic 220 and 400 reflections, whereby peaks at about 51 deg. and 52 deg. could be related to 311 and 222, respectively^36^. The peak at about 48 deg. together with right-hand shoulder on the main peak belong to the unidentified second phase shown up after annealing. With such a tentative approach, the variations of positions of the mentioned peaks towards smaller angles can be interpreted as a manifestation of a large internal compressive state which reduces during annealing. Figure 9 demonstrates a well-pronounced 220 peak ob served already for annealing time of 1 hour so, this wire and as-received one were selected for the comparative study of the physical properties in this work.

The thermomagnetization curves were measured using PPMS (Physical Property Magnetic System) vibrating-sample magnetometer in the temperature range between 5 K and 400 K. A magnetic field, *H*, from 50 Oe to 90 kOe was directed along the sample axis.

A bunch of microwires was used as a sample for the magnetic measurements revealing relative changes of magnetization, which was conveniently presented in terms of the normalized magnetization, *M/M*_*5K*_, where *M* is the magnetic moment at a given H and temperature, *T*, and *M*_*5K*_ is the magnetic moment at *T* = 5K and H = 90 kOe.

Electrical resistance, R, was measured by 4-points method using PPMS option. Magnetic field during the R measurements was applied along the microwire axis.

The magnetoresistance (MR), *ΔR/R*, is defined as:

1 $${\rm{\Delta }}R/{\rm{R}}( \% )=({\rm{R}}({\rm{H}})-{\rm{R}}(0))\times 100/{\rm{R}}(0)$$

The MR was measured for the magnetic field directed along the microwire axis.

The MCE was calculated by a standard Maxwell relationship using measured *M(H)* dependences at different temperatures using the method described by V. K. Pecharsky *et al*.^34^. In this method the field-induced entropy change, *ΔS*, is evaluated by integrating of the following formula:

2 $$\Delta {\rm{S}}{({T})}_{{\Delta }H}={\int }_{HI}^{HF}dS(T,H)={\int }_{HI}^{HF}(\frac{\delta M(T,H)}{\delta T})H\,dH$$

where *HI* and *HF* are the initial and final magnetic field, respectively.

Accordingly, we have measured the virgin magnetization curves *M(H, T)* at different temperatures and then evaluated *ΔS*.

## Conclusions

Our previous efforts of preparation of thin glass-coated microwires of NiMn-based Heusler alloys intended of getting martensitic transformation and related functional properties have been scarcely effective^5–7,10,11^. In the present work, we have successfully prepared Ni_59.2_Mn_12.2_Ga_28.6_ Heusler-type long (up to few meters long) and flexible glass-coated microwires, which exhibit a favorable combination of the magnetic properties with the martensitic transformation behavior. As-prepared microwires present MR effect and considerable dependence of *M(H)* (particularly absolute *M*-values) on the magnetic field attributed to the magnetic inhomogeneities and atomic disorder. Annealing conditions strongly affect the temperature dependence of magnetization and Curie temperature of the as-received microwires. We observed the characteristic hysteretic anomalies in *R(T)* and *M(T)* dependences in the as-prepared and annealed samples produced by the martensitic transformation. Annealing at 973 K, allowing disorder reduction and internal stresses relaxation, gave rise to the magnetic and magnetocaloric properties and transformation behaviour of the microwires qualitatively similar to the bulk alloy with the same e/a = 7.63 characterized by the inequality *T*_*C*_ > *T*_*M*_. On the other hand, *T*_*C*_ and *T*_*M*_ absolute values obtained in the present work do not correspond to the e/a criterion since other factors, such as a considerable off-stoichiometry, structural disorder and high internal stress predominantly control the magnetic and MT properties.

As a final conclusion, in this work we have demonstrated that wire-coated technology is a promising route of obtaining large amount of the quasi-unidimensional FSMAs suitable for applications.
